# Navigating lipid droplet proteins – part II: molecular mechanisms underlying ER-to-lipid droplet protein partitioning

**DOI:** 10.1042/BST20253052

**Published:** 2025-10-29

**Authors:** Louisa Magdalena Krauß, Bianca Schrul

**Affiliations:** 1 Medical Biochemistry and Molecular Biology, Center for Molecular Signaling (PZMS), Faculty of Medicine, Saarland University, Homburg/Saar, 66421, Germany; 2 Center for Biophysics, Saarland University, Germany

**Keywords:** endoplasmic reticulum, intracellular transport, lipid droplets, lipid metabolism, membrane proteins, organelle biogenesis

## Abstract

Lipid droplets (LDs) originate from the endoplasmic reticulum (ER) and are unique among cellular organelles, as they consist of a hydrophobic core of neutral lipids that is surrounded by a phospholipid monolayer. Proteins and enzymes embedded into this monolayer are essential for regulating dynamic lipid storage and consumption and hence, for the cellular adaptation to metabolic changes. Their activity and abundance on the LD surface must therefore be well-controlled. Many of these proteins are first inserted into the phospholipid bilayer membrane of the ER before they partition to the LD monolayer. While a monotopic membrane topology is required for enabling the targeting of these ERTOLD proteins from the ER to LDs, the molecular mechanisms underlying this partitioning are only beginning to emerge. In this second part of the bipartite review ‘Navigating lipid droplet proteins,’ we discuss recent conceptual advances regarding ER-to-LD protein partitioning and focus on novel insights into the structural dynamics of LD-destined proteins, how their partitioning to LDs is temporally controlled, and the hierarchies involved in selective and competitive protein recruitment to LDs according to metabolic needs and functions.

## Introduction – unique physicochemical properties of LDs and their consequences for LD-associated proteins

Lipid droplets (LDs) are key organelles in the regulation of cellular lipid metabolism. They originate from the endoplasmic reticulum (ER) and dynamically manage the storage and the consumption of neutral lipids, thereby fluctuating in size based on cellular demands [1,2]. LD-localized proteins [3–5] are essential to regulate correct LD biogenesis and functions, aberrations of which are implicated in numerous pathologies [6–9]. The molecular mechanisms underlying correct protein targeting to LDs, however, are only beginning to emerge.

**Figure 1:**
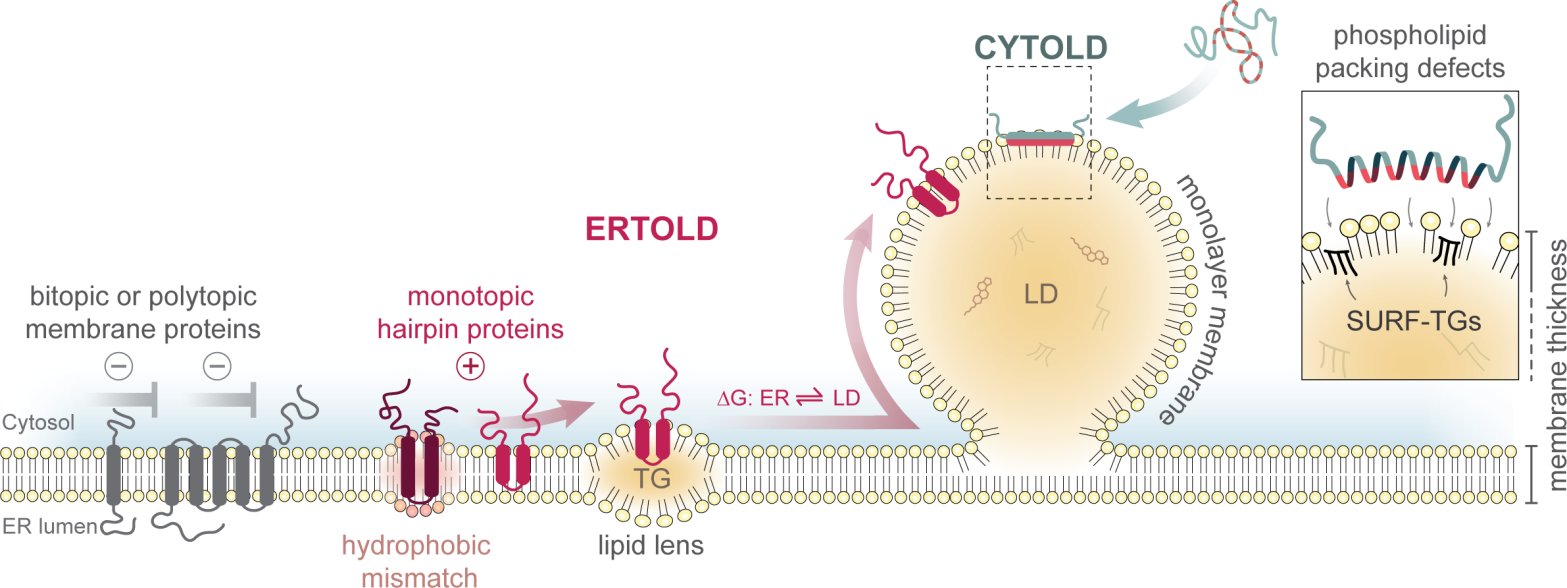
LDs are ER-derived organelles with unique physicochemical properties. The current model of lipid droplet (LD) biogenesis proposes that endoplasmic reticulum (ER)-synthesized neutral lipids, e.g., triglycerides (TGs), accumulate between ER membrane leaflets and coalesce into a lipid lens. Continued neutral lipid synthesis drives LD budding from the cytosolic ER membrane leaflet. A phospholipid monolayer separates the neutral lipid core from the cytosol, with acyl chains embedding in the neutral lipid phase and headgroups facing the cytosol. Packing defects in the LD monolayer, i.e., interfacial voids between phospholipid head groups, transiently expose phospholipid acyl chains and underlying neutral lipids (e.g., SURF-TGs) to the aqueous cytosol. CYTOLD proteins can associate with LDs from a soluble state in the cytosol by, e.g., the aid of amphipathic helices (green/red) that preferentially integrate into such packing defects. Proteins that stably integrate into the monolayer *via* hydrophobic hairpin-like regions reach LDs from the ER membrane (ERTOLD proteins, red), adopting a monotopic topology with soluble domains facing the cytosol in both membrane environments, while transmembrane-spanning proteins with ER lumenal domains (gray) are excluded from the LD monolayer. In a ‘passive lateral diffusion model,’ free energies (ΔG) may act as a driving force for ER-to-LD protein partitioning. A special scenario is a hydrophobic mismatch of, e.g., plant oleosins (dark red), in which the length of the hydrophobic hairpins exceeds ER bilayer thickness, potentially resulting in folding constraints that are relieved upon protein partitioning to the LD monolayer. SURF-TGs, surface-oriented TGs.

In comparison with other organelles, LDs are unique in several aspects: First, LDs are not surrounded by a phospholipid bilayer. Instead, a phospholipid monolayer separates the neutral lipid core [mostly triglycerides (TGs) and sterol esters (SEs)] from the cytosol [1,2,10]. This unique architecture impacts the physicochemical properties of LDs. The hydrophobic core is virtually protein-free, while proteins associate with the surface [11]. Transmembrane-spanning membrane proteins with bitopic or polytopic topologies cannot integrate into the LD surface due to energetic constraints when exposing soluble domains into the hydrophobic neutral lipid core (Figure 1) [12–14]. Consequently, LD proteins either associate with the monolayer peripherally or adopt a monotopic membrane topology, wherein hydrophobic hairpin-like segments or interfacial membrane anchors stably integrate into the monolayer, exposing soluble domains cytosolically [5,15,16]. Second, in contrast with other organelles, there is no dedicated targeting machinery that mediates the direct insertion of LD-destined proteins into its limiting membrane. So-called CYTOLD (for cytosol to LD) are defined as proteins that reach the LD surface from a soluble state in the cytosol, e.g., by directly associating with the LD monolayer with the aid of amphipathic helices or by peripherally engaging with LD-resident proteins. Monotopic hairpin proteins, however, are first inserted into the ER membrane before partitioning to the LD monolayer, defining them as ERTOLD (for ER to LD) proteins [17]. How these ERTOLD proteins are specifically targeted to the ER membrane is discussed in the first part of this bipartite review [18]. Here, we focus on the question of how ERTOLD proteins are then specifically recruited to the LD monolayer.

LDs originate from the cytoplasmic leaflet of the ER bilayer [1,2] (Figure 1) and consequently the LD monolayer consists of this former ER leaflet. The existence of lipid transfer proteins at contact sites [19–21] and phospholipidic enzymes on the LD [22–24], however, suggests further diversification of the composition of the LD monolayer [25,26], while acyl-chain remodeling can influence its fluidity [27,28]. Likewise, the LD monolayer can be less packed with phospholipids than the ER bilayer membrane [29], which can result in higher surface tension and larger and more persistent packing defects in the LD monolayer, with transient exposure of phospholipid acyl chains and the underlying neutral lipid core to the aqueous cytosol [30–33]. Molecular dynamics (MD) simulations suggest that surface-oriented TGs (SURF-TGs) interdigitate into these surface packing defects to reduce interfacial energies and lower surface tension [30,32,34]. Together, packing defects with intercalated SURF-TGs are unique to LDs and can create preferred binding sites for CYTOLD proteins containing amphipathic helices [31,33,34], while the composition of the neutral lipid core becoming surface-accessible within such defects may also affect protein-binding affinities [35,36].

Emerging evidence suggests that similar membrane properties may also influence preferred LD monolayer over ER membrane binding of hairpin-containing ERTOLD proteins [37–40]. Yet, some ERTOLD proteins prefer one compartment over the other, and several ERTOLD proteins do not just transit through the ER to the LD surface but display a functional role in both compartments [41,42]. Hence, their abundance in each organelle must be controlled. While tethering to ER-resident transmembrane proteins can restrict the pool of mobile ERTOLD proteins within the ER [16,41], and distinct physicochemical properties of the ER membrane and LDs provide means for preferred monolayer binding [16], this review emphasizes recent conceptual advances underlying ER-to-LD partitioning beyond these basic principles. This includes novel insights into the structural dynamics of LD-destined proteins, how their partitioning to LDs is temporally controlled, and the hierarchies involved in selective and competitive protein recruitment to LDs according to metabolic needs and functions.

## Stretch or bend? Conformational adaptation of LD-destined proteins during ER-to-LD partitioning

A hallmark characteristic of many ERTOLD proteins is their stable integration into the ER bilayer in a hairpin-like monotopic topology before partitioning to the LD monolayer. These hairpin regions typically consist of two hydrophobic helices, frequently separated by helix-breaking prolines at the hinge, and flanking basic stretches at the membrane-solvent interface, features that often ensure a monotopic membrane topology and allow ER-to-LD partitioning [13,43–46]. In addition, positively charged residues at the hinge region and bulky aromatic residues such as tryptophans at the middle section of the hydrophobic helices seem important to enable efficient LD targeting of ERTOLD proteins [47].

Earlier models proposed that these hairpin regions penetrate only partially into the ER membrane, adopting a conformation that would enable ER-to-LD partitioning via passive lateral diffusion along the cytoplasmic leaflet of the ER membrane when in continuity with the LD monolayer, e.g., during LD biogenesis [5,15,16]. This implies that hairpin ERTOLD proteins are similarly embedded within both ER and LD membranes. Recent findings, however, have challenged this view, revealing that several ERTOLD hairpin proteins are differently positioned in bilayer membranes and LD monolayers [39,47].

**Figure 2:**
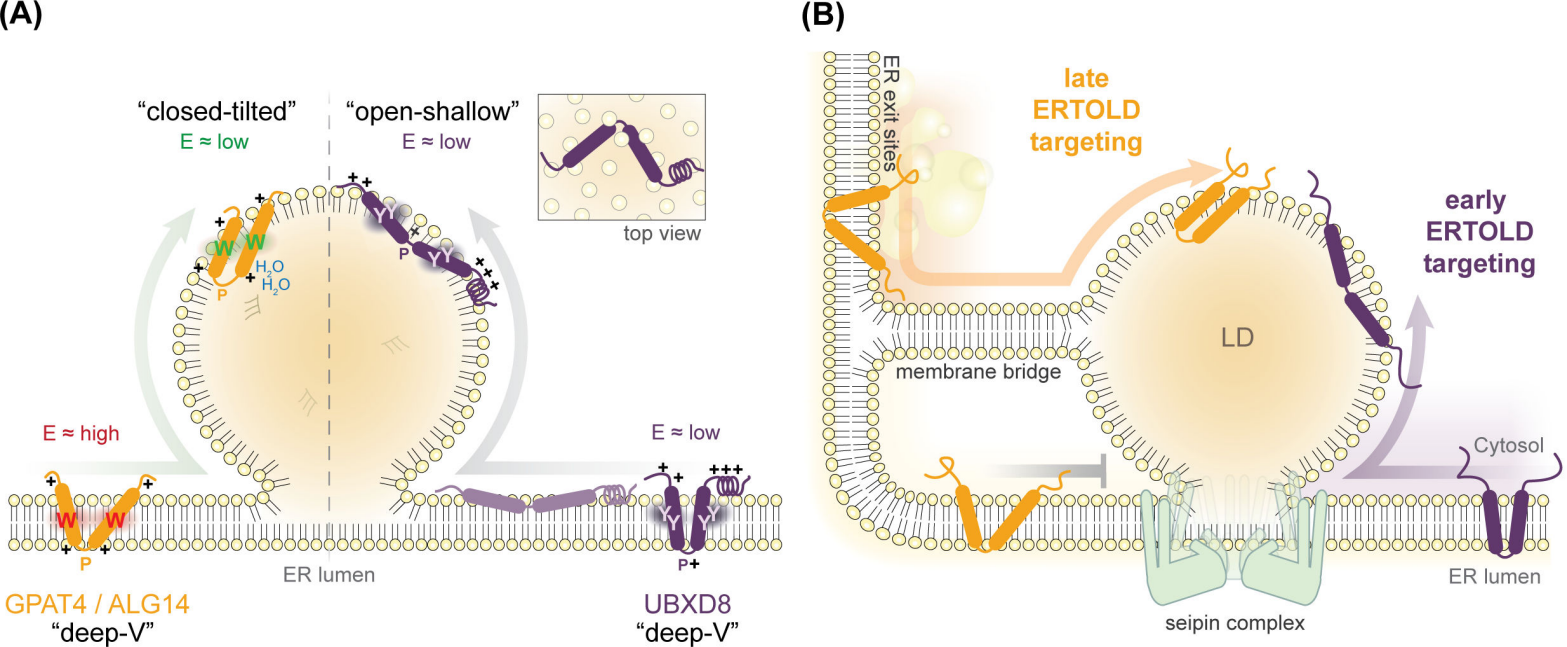
Structural transitions of ERTOLD hairpin proteins are potentially relevant for time-controlled ER-to-LD partitioning. (**A**) ERTOLD hairpin proteins can undergo different structural transitions during ER-to-LD partitioning. GPAT4, ALG14, and UBXD8 embed into the ER membrane in a ‘deep-V’ conformation: A central proline (P) in the hairpin kink region is flanked by at least one positively charged arginine (+), which presumably snorkels to the ER lumenal membrane-solvent interface. Together with positive charges at the cytosolic membrane-solvent interface, these residues may engage in electrostatic interactions that contribute to protein anchoring in a monotopic and deeply inserted configuration. Consequently, large aromatic residues (tryptophans (W) in GPAT4/ALG14; tyrosines (Y) in UBXD8) are positioned near the bilayer midplane. Left: In the LD monolayer, GPAT4/ALG14 adopts a ‘closed-tilted’ conformation, with tryptophans relocating to the cytosolic interface. Likewise, the central proline and one flanking arginine (+) move to the monolayer surface, while the other arginine that was formerly at the lumenal bilayer interface enters the neutral lipid phase, interacting with water (H_2_O) and TG molecules. Free energy calculations suggest that the ‘deep-V’ to ‘closed-tilted’ rearrangement is thermodynamically favorable (E_high_ to E_low_), explaining GPAT4/ALG14 accumulation on LDs [47]. Right: By contrast, UBXD8 adopts an ‘open-shallow’ conformation on the LD surface in which both helices become more solvent-exposed. The kink repositions to the cytosolic face of the monolayer, and the interhelical angle widens relative to the closed deep-V configuration in the bilayer (top view). Free energies required for the ‘deep-V’ to ‘open-shallow’ transition suggest that, in contrast with GPAT4/ALG14, mechanisms beyond thermodynamics govern ER-to-LD partitioning of UBXD8. In addition, MD simulations suggest that the structural transition of UBXD8, either temporarily within the ER bilayer or during the passage to the LD surface, is a prerequisite for efficient partitioning [39]. (**B**) Conceptual model of early and late ERTOLD protein-targeting mechanisms. Early ERTOLD proteins access the LD surface during biogenesis via the seipin-containing LD assembly complex, whereas late ERTOLD proteins are initially restricted and associate later via seipin-independent ER–LD membrane bridges, involving ER exit site components, membrane fusion machinery, and COPI/ARF activity [48]. Temporal segregation may ensure co-ordinated biogenesis, expansion, and turnover of LDs and would restrict premature access of, for example, catabolic enzymes during LD growth. High-resolution molecular structures of the seipin oligomer have been elucidated across multiple species, revealing its multimeric organization into ring-like structures [49–53]. Computational studies have further refined our understanding of seipin function, contributing to a model in which the seipin multimer regulates LD biogenesis through conformational flexibility of its transmembrane regions within the ER bilayer [54–56]. An initial closed conformation of the seipin ring structure condenses neutral lipids below a critical threshold. Upon reaching this threshold, a conformational switch repositions the transmembrane segments into an open state, promoting LD expansion and cytosolic budding [57–59]. Seipin’s open state may act as a selective gatekeeper for membrane proteins, allowing passage of shallowly embedded proteins while restricting deeply inserted ones. ER, endoplasmic reticulum; LD, lipid droplet; GPAT4, glycerol-3-phosphate acyltransferase 4; ALG14, UDP-N-acetylglucosaminyltransferase subunit; UBXD8, UBX domain-containing protein 8; MD, molecular dynamics; COPI, coat protein complex I; ARF, ADP-ribosylation factor;

MD simulations indicate that the membrane-embedded domains of the fly proteins glycerol-3-phosphate acyltransferase 4 (GPAT4) and UDP-N-acetylglucosaminyltransferase subunit (ALG14) [47] and of mammalian UBX domain-containing protein 8 (UBXD8), [39] deeply integrate into the ER membrane with positively charged residues in the hairpin kink region anchoring them at the lumenal membrane-solvent interface through electrostatic interactions with phospholipid headgroups (‘Deep-V’ conformation in Figure 2A). Upon transitioning to the LD surface, these proteins undergo a structural rearrangement. However, while GPAT4/ALG14 adopts a ‘closed-tilted’ conformation on the LD surface [47], UBXD8 transitions to an ‘open-shallow’ conformation with its two hydrophobic helices splaying outward to form a wide interhelical angle [39] (Figure 2A).

For GPAT4/ALG14, the transition from a ‘deep-V’ to a ‘closed-tilted’ state appears a thermodynamically driven repositioning, facilitating an energetically more favorable conformation within the LD monolayer [47]. A similar concept has been proposed earlier for plant oleosins, which integrate into the ER bilayer with rather long hydrophobic hairpins presumably leading to a hydrophobic mismatch in the ER [60,61] with folding constraints [62] that are relieved upon protein partitioning to the LD monolayer [63] (Figure 1).

In contrast, for UBXD8, both the ‘closed deep-V’ conformation in the ER and the ‘open-shallow’ positioning on the LD surface appear stable and long-lived, and the transitioning between these states involves a high energy barrier, suggesting that this process is likely not spontaneous. Interestingly, MD simulations indicate that only UBXD8 molecules in their ‘open-shallow’ conformation can efficiently partition from the bilayer to the monolayer [39], raising the question of which factors in the ER membrane or during the passage through the ER–LD neck drive the structural rearrangement (Figure 2A).

Analogously to the hydrophobic mismatch of oleosins in the ER bilayer, it is conceivable that locally increased bilayer thickness may render the ‘deep-V’ conformation of UBXD8 energetically unfavorable, thereby promoting the transition to the ‘open-shallow’ conformation. Likewise, local alterations of membrane curvature or tension, surface charges, additional proteins, or a combination thereof might influence the intramembrane positioning of UBXD8. Currently, the molecular membrane architecture of LD biogenesis sites is not well understood. But interestingly, UBXD8 appears to target the LD early during its biogenesis, while GPAT4 only partitions there later [48], raising the possibility that different conformational changes in these proteins are mechanistically relevant for time-controlled ER-to-LD partitioning.

## Now or later? Temporal coordination of ER-to-LD protein partitioning

ERTOLD proteins exhibit distinct spatiotemporal kinetics and partition to LDs at different stages within the LD life cycle. Some proteins, such as long-chain acyl-CoA ligase 3 (ACSL3) [64,65], LD assembly factor 1 (LDAF1) [66,67], LD-associated hydrolase (LDAH), UBXD8, and the synthetic model peptide LiveDrop, derived from GPAT4 [47,48,68], associate with nascent LDs during early stages of LD formation. In contrast, full-length GPAT4 [47,48,69], LD subset dehydrogenase 1 (LDSDH1), and 17-beta-hydroxysteroid dehydrogenase 11 (HSD17B11) are initially excluded from nascent LDs and only localize to LDs upon maturation [48]. Mechanistically, early and late ERTOLD protein targeting follow two distinct pathways [48] (Figure 2B).

Early-targeting cargoes partition to the surface of nascent LDs, presumably by traversing the ER-localized LD assembly complex at the ER–LD neck [70–72]. These complexes define LD formation sites and contain multimeric ring-like assemblies of the ER-resident transmembrane protein seipin and associated cofactors [49,57–59,66,67,73]. Single-molecule tracking experiments have substantiated the role of LD assembly complexes as principal entry points for early ERTOLD cargoes [74]. Here, seipin has been proposed to function as a selective barrier, enabling early ERTOLD proteins to reach the LD surface while temporarily restricting LD access to late ERTOLD proteins during LD formation. The aberrant, premature LD localization of late-targeting cargoes in the absence of seipin supports this role [48,68], but the molecular details underlying its gatekeeping function remain speculative. One possibility is that the seipin complex, via its transmembrane segments spanning the ER membrane in its proposed open configuration during LD emergence [50,51], may act as a molecular sieve, sterically excluding certain proteins from LD association while permitting the passage of structurally compatible cargoes (Figure 2B). Alternatively, seipin may not only restrict access but also actively recruit a subset of proteins to the LD surface during early timepoints. This could involve modulation of the membrane microenvironment at the ER–LD neck [29,38,58] or the induction of protein conformational changes that prime cargoes for early LD targeting. Both hypotheses would align with the reported conformational change of the early-targeting protein UBXD8 from a ‘deep-V’ conformation to an ‘open-shallow’ state upon LD association [39]. Proteins undergoing such structural rearrangement could be exceptionally qualified to pass the seipin barrier, while such rearrangement could even be facilitated by seipin, scenarios that need to be addressed in future studies.

Given that late-targeting cargoes may not be able to traverse the seipin barrier at the ER–LD neck [48], alternative routes must exist for their recruitment to LDs. Indeed, fluorescence recovery after photobleaching analyses indicate that GPAT4 circumvents seipin-containing LD assembly complexes for late LD localization and may instead utilize membranous ER–LD continuities [48,69] (Figure 2B). Genome-wide RNAi screening in flies identified key regulators of late ERTOLD targeting, particularly genes involved in membrane fusion and tethering processes and ER exit site organization, as well as COPI vesicle machinery [48] that has been implicated in LD–ER bridge formation before [75]. Silencing these genes abolished LD localization of late ERTOLD cargoes, causing their retention in the ER membrane, whereas early ERTOLD cargoes and CYTOLD proteins remained unaffected [48]. Despite requiring ER exit site components, late ERTOLD targeting appears to operate independently of their canonical role in anterograde vesicular transport along the secretory pathway [48], which is consistent with motion dynamics of the late ERTOLD protein GPAT4 rather reflecting diffusion than vesicular trafficking kinetics [69,75]. Several of the identified late-targeting machinery factors localized to ER sites adjacent to LDs, with their association increasing during later stages of LD maturation [48]. Together, these observations led to the hypothesis that the formation of physical ER–LD membrane continuities, distinct from seipin-mediated contacts, facilitates late ERTOLD cargo recruitment [48,69,75]. The molecular mechanisms underlying the establishment of such membrane bridges remain largely elusive, but a preliminary working model outlining the sequence of events has recently been discussed in detail by Klemm and Carvalho [1].

The concept of seipin-independent ER–LD membrane bridges as conduits for late ERTOLD cargo transport remains debated, and an alternative hypothesis proposes that rather than establishing new ER–LD membrane bridges, late-targeting machinery may instead modulate the surface properties of mature but still seipin-connected LDs, creating an environment more permissive for late-targeting cargoes [59]. This hypothesis would be consistent with the observation that seipin-defined gateways persist beyond initial LD formation, also serving as exit sites to facilitate protein relocalization to ER [74]. Resolving these possibilities will require more high-resolution structural insights into the architecture and establishment of the proposed ER–LD membrane bridges during late LD life cycle phases. In any case, the temporal co-ordination of protein targeting likely plays a crucial role in LD biogenesis, preventing surface overcrowding and ensuring that lipid-modifying enzymes reach LDs at appropriate maturation stages [48].

## First come, first served? Hierarchical dynamics in crowding and competition during ER-to-LD protein partitioning

The coarse categorization into early and late ERTOLD pathways, as outlined above, is likely insufficient to fully explain the intricate and dynamic LD proteome remodeling required during metabolic adaptations and does not explain how differential recruitment of proteins within one of these categories is achieved. Here, the availability as well as the physicochemical properties of the LD surface area becomes decisive determinants.

**Figure 3:**
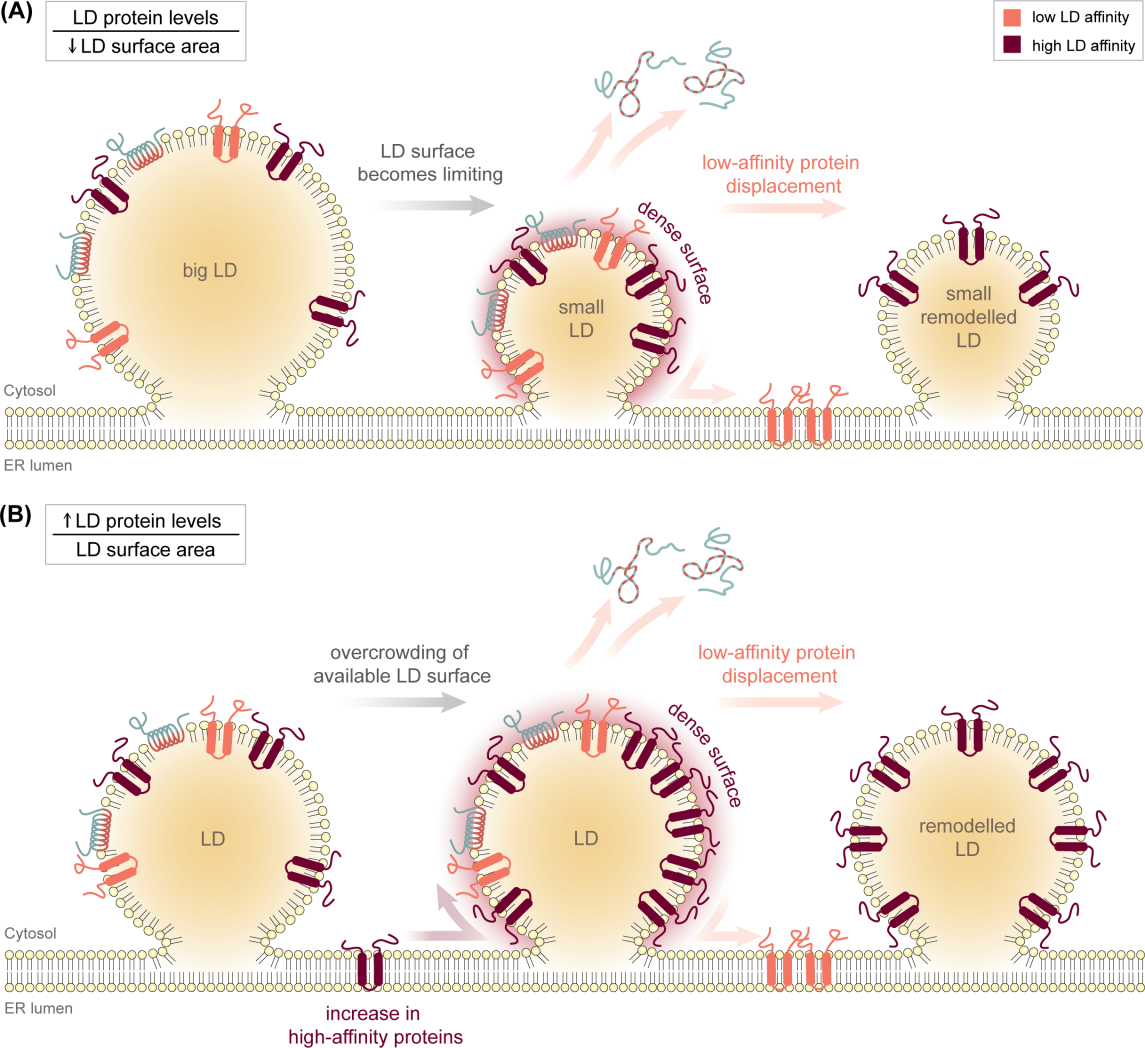
Protein crowding and competition on the LD surface. (**A**) Dynamics in lipid droplet (LD) size affect proteome remodeling. Under catabolic conditions, neutral lipids in the LD core undergo hydrolysis to fuel metabolic processes, which leads to progressive shrinkage of LDs. Consequently, the limiting LD surface area results in protein crowding and competition for lipid-binding sites. Proteins with lower binding affinities (light-red hairpins and blue/red amphipathic helices) are gradually displaced from the LD monolayer to the cytosol, or they relocate to the ER membrane. Conversely, during LD growth, additional lipid-binding sites on the LD surface become available, enabling the association of additional proteins (not shown). (**B**) Changes in protein abundance lead to LD proteome remodeling. Even if the LD surface area remains constant, increased expression of high-affinity proteins (dark-red hairpins) can lead to LD proteome remodeling. As an example, increased expression of PLIN1 during adipocyte differentiation can lead to displacement of lower affinity PLIN family members, finally demarcating distinct LD subpopulations. ER, endoplasmic reticulum; PLIN, perilipin

Limiting the LD surface area, for example, during shrinkage of LDs under catabolic conditions, leads to macromolecular surface crowding and presumably induces lateral collisions among LD-associated proteins [76] (Figure 3A). Simultaneously, biophysical surface properties change; most notably, the surface tension decreases [34,77], and surface packing defects and exposure of SURF-TGs are reduced [30,34]. Consequently, LD-associated proteins must compete for a diminishing number of binding sites, leading to a gradual displacement of proteins with weaker membrane affinities [15,76] (Figure 3A). Among these proteins, amphipathic helix-containing proteins, which associate with the LD surface by intercalating bulky hydrophobic residues from their hydrophobic helix interfaces into large surface packing defects [17,31,34,35], appear particularly susceptible to displacement. In contrast, proteins with larger hydrophobic domains, such as hairpin-containing ERTOLD proteins, may exhibit greater retention [76], although relocation of ERTOLD proteins from the LD surface to the ER membrane has been observed [74,78,79]. While macromolecular crowding-dependent competition among LD proteins is particularly pronounced during catabolic LD shrinkage, the LD surface availability may also become limiting upon elevated expression of high-affinity proteins, leading to reduced LD association of proteins with lower LD affinities (Figure 3B) [40,76,80].

The hierarchical binding dynamics of LD proteins during LD emergence are exemplified by the perilipin (PLIN) protein family, comprising five evolutionarily related PLIN proteins that abundantly localize to LDs [81,82]. Despite their overall similar domain organization, PLINs exhibit different LD-binding affinities and demarcate different LD subpopulations within a cell. Historically, PLINs have been considered canonical CYTOLD proteins, associating with the LD surface from a soluble state in the cytosol via their conserved amphipathic N-terminal 11-mer repeat motifs [83–87], while C-terminal four-helix bundles [88] presumably modulate this amphipathic-helix-mediated LD binding [77]. The collective interplay of several protein domains and PLIN isoform-specific alterations in these domains likely accounts for their differential LD affinity [77,81]. Interestingly, however, an additional integral membrane segment in PLIN1 mediates its insertion into the ER membrane [80], reclassifying PLIN1 as a *bona fide* ERTOLD protein [89].

Consistent with the idea that ERTOLD proteins are dominant over CYTOLD proteins when competing for the LD surface, PLIN1 shows the highest LD affinity among PLIN proteins and effectively displaces the others [40,77,80]. This would explain that during adipocyte differentiation, PLIN1 saturates existing LDs as long as ER-localized PLIN1 pools are sufficient. As differentiation progresses and these ER-resident pools could become exhausted, other PLIN isoforms gain access to newly emerging LDs via the CYTOLD pathway. This model would be consistent with the observation that PLIN2, PLIN3, and PLIN4 require a transient increase in LD surface tension for effective binding, as expected for more rapidly emerging LDs [33]. Such hierarchical ERTOLD over CYTOLD protein binding and sequential LD formation would explain the existence of distinct LD subpopulations with separate protein pools [1,40,80]. But which parameters determine the hierarchies among hairpin-containing ERTOLD proteins?

In an elegant *ex-cellulo* approach, Damm and colleagues recently determined the ER-to-LD partition coefficients for a range of ERTOLD proteins that were either individually expressed or co-expressed in pairs [40]. Proteins with higher LD affinity competitively displaced those with lower affinity. While this experimental system does not yet fully recapitulate the ER-to-LD partitioning dynamics observed in intact cells for all ERTOLD proteins, it proves powerful in systematically assessing competition between ER proteins for their relocation to LDs and suggests that steric repulsion is decisive in fine-tuning the LD proteome. In addition, it suggests that active prevention of premature ER-to-LD partitioning of higher-affinity proteins, for example, by a seipin-mediated gatekeeper function, is important to prioritize efficient LD binding of lower-affinity proteins early during LD emergence. These could be gradually displaced from the LD surface once higher affinity proteins, potentially targeting the LD via the late ERTOLD pathway, compete for the available surface space [40].

## Rooted in the core – impact of neutral lipid core properties on LD proteome remodeling

In addition to the different structural dynamics between ERTOLD proteins, the temporal control of ER-to-LD partitioning, and the hierarchies involved in protein crowding on the LD surface, the composition of the neutral lipid core can also influence differential protein association with LDs. TG and SE are the primary constituents of the neutral lipid core in LDs across many cell types, with their relative abundances varying by tissue and metabolic state [26].

TG-rich LDs are commonly found in metabolically active cells like adipocytes, hepatocytes, and muscle cells, while SE-rich LDs are more prevalent in steroidogenic cells, including ovarian granulosa or adrenocortical cells [28,90,91]. LDs isolated from cells capable of forming both LD types, such as naïve rat granulosa cells [92] or THP-1 macrophages [93], exhibit significant proteomic differences depending on substrate-specific treatments to induce either TG-rich or SE-rich LDs, suggesting that the LD core composition influences the LD proteome. Importantly, differential isolation of SE- and TG-rich LD subpopulations from mouse adrenocortical cells revealed LD subtype-specific sequestration of PLIN protein family members. This provides evidence that the lipid core composition directly affects protein recruitment to LDs independent of transcriptional changes or posttranslational modifications in response to different feeding stimuli [36].

**Figure 4:**
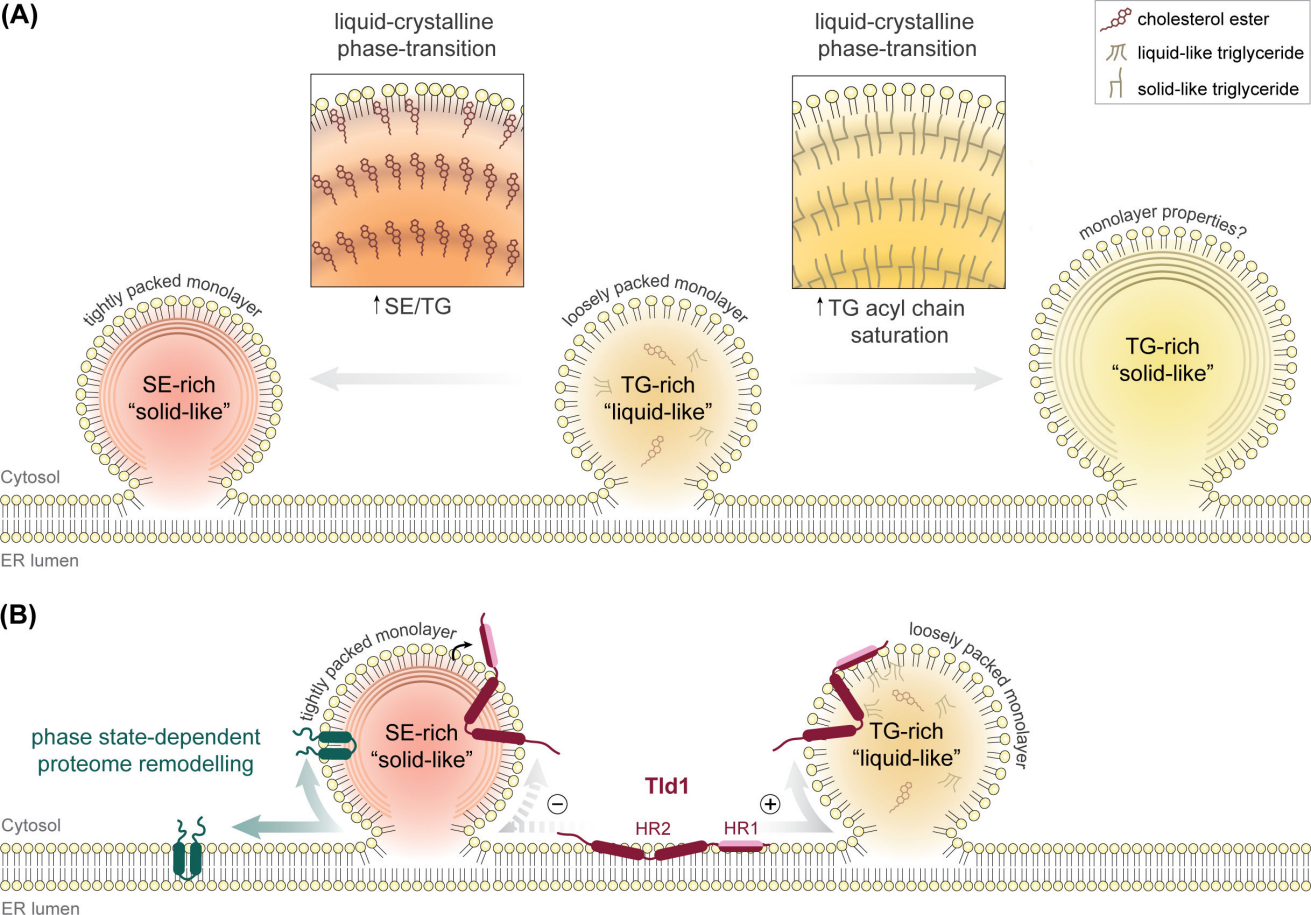
LD core phase transitions and altered physicochemical properties of the LD monolayer can affect differential protein targeting to LD subpopulations. (**A**) Physicochemical properties of lipid droplets (LDs) upon phase-state transition: The neutral lipid core of LDs can transition between ‘liquid-like’ amorphous and ‘solid-like’ smectic crystalline phases, either driven by increasing SE/TG ratios, as observed under starvation conditions (left; [97,98]), or by increased TG incorporation and acyl chain saturation, as seen in adipose tissues of high-calorie diet-fed mice (right; [99]). In the ‘solid-like’ state, SE (left) or TG (right) molecules form tightly packed concentric layers at the core periphery. MD simulations indicate that in SE-rich LDs, SE molecules intercalate into the phospholipid tail region of the LD monolayer [97,100], resulting in a more tightly packed monolayer with higher phospholipid ordering, fewer packing defects, and fewer SURF-TGs [100]. It remains to be investigated whether ‘solid-like’ TG-rich LDs (right) exhibit comparable alterations in monolayer properties. (**B**) LD proteome remodeling upon phase-state transition: Phase-state transition in the LD core can lead to remodeling of the LD surface proteome [97] as schematically illustrated by a representative ERTOLD protein depicted in green (left). An attractive model based on MD simulations proposes that proteins, such as Tld1, depend on interactions with SURF-TGs to interact with TG-rich LDs and are displaced from SE-rich LDs [101]. Tld1 contains two membrane-associated regions: an amphipathic helix (HR1) for LD localization and a hydrophobic hairpin region (HR2) favoring ER localization. MD simulations suggest a membrane property-sensing mechanism underlying Tld1’s core-dependent LD localization: In the ER membrane, HR2 inserts shallowly into the cytoplasmic membrane leaflet by adopting a wide-angle conformation, stabilized by interactions between polar residues within the hairpin kink region and phospholipid glycerol moieties. Upon LD association, the kink region penetrates the LD interior, interacting with the neutral lipid core and closing the hairpin angle. While HR1 integrates into surface packing defects present on TG-rich LDs, it does not incorporate into the surface of more densely packed SE-rich LDs, providing specificity for Tld1 targeting to TG-rich LDs (+) and excluding it from SE-rich LDs (−) [101]. SE, sterol esters; TG, triglycerides; MD, molecular dynamics; Tld1, TG-associated LD protein 1; HR, hydrophobic region;

The molecular mechanisms underlying the biogenesis of distinct LD subpopulations [94,95] and how their subtype-specific proteomes are shaped by differences in neutral lipid core composition remain subjects of ongoing investigations (reviewed in detail in [96]), but alterations in their physicochemical properties likely affect protein association. Indeed, high-resolution cryo-electron tomography of yeast and mammalian cells revealed that the interior of LDs containing a mixture of SE and TG undergoes a phase transition as the ratio of SE/TG increases, such as under starvation conditions with elevated TG lipolysis [97,98] or during mitotic arrest [98]. Under these conditions, the amorphous liquid phase of the lipid core reorganizes into smectic liquid crystalline lattices, in which SE molecules align in stacked concentric layers around an amorphous TG core, resembling an onion’s cross-section (Figure 4A, left) [97,98]. Such phase transitions in the neutral lipid LD core were also reported in human hepatocytes derived from non-alcoholic fatty liver disease patients ([102] and citations therein), suggesting disease-associated lipid packing signatures.

Albeit less characterized than SE-rich phase transitions, similar phenomena were reported in mouse adipose tissue upon a high-calorie diet, which results in elevated TG/SE ratios and higher TG acyl chain saturation [99]: under these conditions, TGs are tightly packed in ordered crystalline structures, arranged in stacked lamellar layers around a low-order liquid-like TG core (Figure 4A, right), a property that correlates with increased LD rigidity and adipose tissue stiffness in high-calorie-fed mice [99].

While systematic proteomic analyses of LDs with varying crystalline core states are required to globally assess LD proteome remodeling, such phase transitions appear to directly affect selective recruitment of ERTOLD proteins: in yeast, several ERTOLD proteins, including Erg6, relocate to the ER during glucose restriction, which induces TG lipolysis and neutral lipid phase transition. Heating to reverse the phase transition restored protein localization to the LD surface, suggesting that indeed phase transitions drive LD proteomic changes rather than lipid composition alterations [97]. MD simulations suggest that SE molecules accumulate at the phospholipid surface, intercalating their cholesterol moieties into the acyl chain layer and altering surface properties (Figure 4A, left). The decrease in packing defects and higher order within the monolayer upon phase transition [97,100] could therefore displace proteins that depend on surface-exposed TGs from SE-rich LDs [101].

Indeed, MD simulations propose that TG-associated LD protein 1 (Tld1) adopts distinct intramembrane conformations in ER bilayers and LD monolayers, influenced by the neutral lipid core composition. One of its two membrane-associated regions forms an amphipathic helix that potentially acts as a sensor for surface packing defects. As these are reduced in SE-rich LDs, helix integration into the monolayer is prevented, displacing Tld1 from SE-rich LDs [101] (Figure 4B). The yeast LD organization protein Ldo16 shares domain architecture with Tld1 [103,104] and colocalizes with Tld1 on specific LD subpopulations [105] that appear protected from lipolysis [103,104,106]. It will be interesting to test whether the sensing mechanism suggested for Tld1 is conserved among other LD-subpopulation-specific proteins, as this could be an underlying concept for how heterogeneity of the LD proteome and, consequently, their metabolic fate is established. Notably, MD simulations suggest that the CYTOLD protein Max-like protein X (MLX) employs a similar mechanism, in which amphipathic helices recognize shallow packing defects on the LD monolayer that expose phospholipid acyl chains [107]. As these are comparatively scarce on SE-rich LDs [100], the model provides a possible explanation for the preferential localization of MLX to TG-rich LDs [93].

**Perspectives**

- Lipid droplets (LDs) are key organelles in co-ordinating cellular lipid metabolism, and aberrations in their biogenesis or dynamics are associated with a plethora of diseases. This includes the correct recruitment of metabolically active or regulatory proteins from the endoplasmic reticulum (ER) membrane to the LD monolayer (ERTOLD proteins).
- ERTOLD proteins are first inserted into the phospholipid bilayer membrane of the ER in a monotopic topology before they can partition to the LD monolayer. While this monotopic topology appears essential for enabling the transitioning from the ER to LDs, the molecular mechanisms underlying this process involve distinct physicochemical properties of the ER and LD membranes, structural properties of ERTOLD proteins, and proteinaceous regulatory factors.
- While several principles and components enabling ER-to-LD protein partitioning have recently been revealed, their interplay and the hierarchies among them are only beginning to emerge and need to be addressed in future studies to understand how LD functions are regulated in healthy and disease states. Likewise, while new concepts regarding the molecular nature of protein–LD interactions unfold, key mechanistic questions about protein insertion depth into LDs, transient intramembrane positioning, and energetics involved in distinct protein–lipid interactions are often only addressed by MD simulations and await experimental verification.

## Abbreviations

ACSL3: long-chain acyl-CoA ligase 3
ALG14: UDP-N-acetylglucosaminyltransferase subunit
ARF: ADP-ribosylation factor
COPI: coat protein complex I
ER: endoplasmic reticulum
GPAT4: glycerol-3-phosphate acyltransferase 4
HSD18B11: 17-beta-hydroxysteroid dehydrogenase 11
LD: Lipid droplet
LDAF1: lipid droplet assembly factor 1
LDAH: lipid droplet-associated hydrolase
LDSDH1: lipid droplet subset dehydrogenase 1
MD: molecular dynamics
MLX: max-like protein X
PLIN: perilipin
SE: sterol ester
SURF-TG: surface-oriented TG
TG: triglyceride
Tld1: TG-associated LD protein 1
UBXD8: UBX domain-containing protein 8
